# Factors Associated with Callus Formation in the Plantar Region through Gait Measurement in Patients with Diabetic Neuropathy: An Observational Case-Control Study

**DOI:** 10.3390/s20174863

**Published:** 2020-08-28

**Authors:** Ayumi Amemiya, Hiroshi Noguchi, Makoto Oe, Kimie Takehara, Yumiko Ohashi, Ryo Suzuki, Toshimasa Yamauchi, Takashi Kadowaki, Hiromi Sanada, Taketoshi Mori

**Affiliations:** 1 Department of Nursing Physiology, Graduate School of Nursing, Chiba University, 1-8-1 Inohana, Chuo-ku, Chiba-shi, Chiba 260-8672, Japan; 2 Department of Smart Sensing Technology, Electrical and Information Engineering, Faculty of Engineering, Osaka City University, 3-3-138 Sugimoto Sumiyoshi-ku, Osaka 558-8585, Japan; hnoguchi@osaka-cu.ac.jp; 3 Department of Clinical Nursing, Faculty of Health Sciences, Institute of Medical, Pharmaceutical and Health Sciences, Kanazawa University, Ishikawa 920-0942, Japan; moe-tky@umin.ac.jp; 4 Department of Nursing, Graduate School of Medicine, Nagoya University, 1-1-20 Daiko-Minami, Higashi-ku, Nagoya City, Aichi 461-8673, Japan; kwaku-tky@umin.ac.jp; 5 Department of Nursing, The University of Tokyo Hospital, 7-3-1 Hongo, Bunkyo-ku, Tokyo 113-8655, Japan; ohyumi-tky@umin.ac.jp; 6 Department of Diabetes Endocrinology and Metabolism, Tokyo Medical University, 6-1-1 Shinjuku, Shinjuku-ku, Tokyo 160-8402, Japan; ryosuzuki-tky@umin.ac.jp; 7 Department of Diabetes and Metabolic Diseases, Graduate School of Medicine, The University of Tokyo, 7-3-1 Hongo, Bunkyo-ku, Tokyo 113-0033, Japan; tyamau-tky@umin.net (T.Y.); kadowaki-3im@h.u-tokyo.ac.jp (T.K.); 8 Department of Gerontological Nursing/Wound Care Management, Graduate School of Medicine, The University of Tokyo, 7-3-1 Hongo, Bunkyo-ku, Tokyo 113-0033, Japan; hsanada-tky@umin.ac.jp; 9 Global Nursing Research Center, Graduate School of Medicine, The University of Tokyo, 7-3-1 Hongo, Bunkyo-ku, Tokyo 113-0033, Japan; 10 Next Generation Artificial Intelligence Research Center, The University of Tokyo, 7-3-1 Hongo, Bunkyo-ku, Tokyo 113-0033, Japan; tmoriics-tky@umin.ac.jp

**Keywords:** diabetic foot, diabetic neuropathies, foot, footwear characteristics, lower extremity joint angles, shear stress, shoes, pressure, prevention of callus formation, walking

## Abstract

Callus has been identified as a risk factor leading to severe diabetic foot ulcer; thus, it is necessary to prevent its formation. Callus formation under the first, second, and fifth metatarsal heads (MTHs) is associated with external forces (pressure and shear stress) during walking. However, the gait factors increasing the external forces remain undetermined. Thus, this study aims to identify the factors increasing the external forces to prevent callus formation. In 59 patients with diabetic neuropathy wearing their usual shoes, the external forces, and the lower extremity joint angles were measured using MEMS force sensors and motion sensors. The external forces and their relationship with the lower extremity joint angles and footwear size were determined. Risk factors causing high external forces on the first MTH included small flexion of the knee joint (*p* = 0.015) and large ankle pronation motion (*p* = 0.034) to obtain propulsion. For the second MTH, wearing excessively long footwear was identified (*p* = 0.026). For the fifth MTH, high external force was related to tight width footwear (*p* = 0.005). An effective intervention for preventing callus formation for the first MTH would involve assisting the push-off foot motion using rocker-sole footwear or gait training. For the second and fifth MTHs, wearing appropriate size footwear would be effective.

## 1. Introduction

Callus formation may lead to the development of foot ulcers and involves hyperkeratosis caused by excessive mechanical loading [1,2,3]. In over 82% of patients with diabetic foot ulcers, callus formation has been found to precede ulcer formation [4]. The relative risk of an ulcer developing under an area of callus has been reported to be 11.0 compared with that under an area without callus [5,6]. Once a foot ulcer occurs, treatment is difficult and takes considerable time [7], making prevention the better strategy for clinical management. Additionally, 58.2% of community-dwelling older adults had corn and callus [8]. Even healthy people with mechanical hyperkeratotic lesions (callus) showed a decrease in Quality of Life (QoL) [9]. Thus, it is necessary to prevent callus formation.

Although callus management commonly involves removing keratinized layers with a corn cutter or scalpel [10,11], callus was found to recur in 84% of patients with diabetes [12]. Tailor-made footwear for callus prevention is available, with demonstrated effectiveness [13,14]. However, Scirè et al. found that the callus recurred in 41% of patients—even if they wore therapeutic footwear [12]. Current interventions for callus are performed as symptomatic treatment: they do not attempt to remove the cause.

The causes of callus formation have not been completely identified. In a study, we determined that callus formation in the first and second metatarsal heads (MTHs) was associated with high shear stress—time integral/pressure time integral (SPR-i). Meanwhile, callus formation in the fifth MTH was associated with high peak shear stress (PSS) [15].

It is necessary to identify modifiable gait characteristics to prevent foot complications in patients with diabetes. The magnitude of lower extremity joint angles as a modifiable gait characteristic is related to the load applied to the foot. However, that relationship has not been examined in the field of diabetic foot prevention. A new area of focus involves not only pressure and shear stress but also the relationship between them and lower extremity joint angles.

In this study, lower extremity joint angles were the focus. The lower extremity joint angles were defined as the motion of the hip, knee, and ankle joints. It was considered that the general gait variables (e.g., cadence, and step length) may not completely reflect the specific gait associated with callus formation. Studies examining the gait of subjects with callus have considered cadence and step length [16]. We compared the data of subjects in two studies, subjects with callus and slow-walking subjects without callus; based on our findings, both were found to have similar values of gait variables [16,17]. Patients with diabetic neuropathy have been observed to have a slow walking speed, small stride, and large gait variability [18]. These factors have often been attributed to the loss of sensation and impaired neuromuscular control. Because of the many similarities in the gait between patients with diabetes neuropathy and those with callus, many patients with callus are probably included among patients with diabetic neuropathy. However, the existence of callus was not found to differ significantly according to the presence or absence of diabetes [19]. Therefore, we speculated that these general gait variables are not reflected in the specific gait of patients with callus. The motion of the hip, knee, and ankle joints were researched in a study as a gait characteristic [20]. We focused on it, because the magnitude of lower extremity joint angles as a modifiable gait characteristic is found to be related to external forces (pressure and shear stress) rather than other parts of the body. The relationship between callus formation and these gait characteristics has not been investigated.

Hitherto, the relationship between pressure and shear stress associated with callus formation and lower extremity joint angles also has not been examined. Thus, it is necessary to evaluate that relationship to find a way to reduce external forces. Furthermore, footwear characteristics, such as shoe size, may be closely related to external forces. Accordingly, the relationship between pressure and shear stress associated with callus formation and lower extremity joint angles and footwear characteristics must be clarified. To clarify these relationships, measurements were performed using a new external force sensor based on MEMS (Micro Electro Mechanical Systems) technology and a motion sensor consisting of an acceleration sensor and an angular velocity sensor.

This study aimed to guide future research or clinical practice in reducing callus formation. Thus, the external forces associated with callus formation and their relationship with lower extremity joint angles and footwear characteristics by studying the gait of patients with diabetic neuropathy are elucidated.

## 2. Materials and Methods

### 2.1. Research Design

The research was conducted as an observational case-control study.

### 2.2. Participants and Procedures

This survey was conducted at the Diabetic Foot Outpatient Clinic at the University of Tokyo Hospital from April to October 2015. The inclusion criteria were diabetic neuropathy patients aged ≥20 years who could walk without aid. Most of the patients with foot ulcers are elderly people. Therefore, this study only targeted adults. We excluded participants who had current diabetic foot ulcers or history of lower limb amputation proximal to the metatarsophalangeal (MTP) joint. Sixty-four patients with diabetes visited the Diabetic Foot Outpatient Clinic, and 59 patients with diabetic neuropathy were included in the survey.

The survey protocol was approved by the ethical committee of the Graduate School of Medicine, The University of Tokyo (#10797). After general examination at the Diabetic Foot Outpatient Clinic (including regular callus removal), written informed consent was obtained from all participants. First, body weight, foot size and width (standing position), footwear characteristics, and toe-gap forces (muscle strength in the lower limb [21]) were measured. Foot width was measured between the first and fifth MTHs. Second, force and motion sensors were attached, and the external forces and lower extremity joint angles were measured during walking. As practice, the patient walked approximately 50 m to confirm that there was no pain and no interference when walking with the sensor attached to their own shoes with standardized socks. For the measurement, the patients walked approximately 15 m in a flat corridor twice. The data of more than 12 steps were used for the analysis in reference to a previous research [22]. An assistant recorded the 15-m walking time using a stopwatch for calculating walking speed and walked behind the patient to prevent them from falling or stop adverse events.

### 2.3. Data Collection

#### 2.3.1. Callus

Callus was defined as a plate-shaped hyperkeratosis observed by a certified nurse of Diabetes Nursing and a Certified Diabetes Educator who are experts in foot care. They had more than 10 years of foot care experience. The presence or absence of callus formation was checked again after 1 month in all patients: generally, the callus recurs within 1 month. First, all calluses were removed. A region having such a callus was classified as the callus formation group if the callus recurred. If the callus did not recur in that region, we excluded it from the analysis. The region without callus was classified as the non-callus formation group if the callus did not develop after 1 month. If the callus developed in the region without callus within the 1-month follow-up, we excluded it from the analysis: we did so since it could not be determined whether the measured gait and foot condition were related to callus formation.

#### 2.3.2. External Forces

The external forces sensor (ShokacChip™; Touchence Inc, Tokyo, Japan) is a tactile sensor with small–high sensitivity based on MEMS technology. In this study, pressure and shear stress in the first, second, and fifth MTHs were measured using the method developed in our previous study [23]. The reliability and validity of this method have already been confirmed. In a single measurement, only two regions could be measured per foot; this is because a maximum of four sensors per foot could be used owing to hardware restrictions of the sensor circuit system. We preferentially selected the callus regions for measurement, and the non-callus regions were selected at random in the three regions.

The peak plantar pressure, pressure time integral, PSS, and shear stress integral of each gait cycle were calculated using the recorded force profile [24,25,26]. In addition, the shear stress–pressure ratios (SPRs) were calculated by dividing shear stress by pressure, that is, peak values (SPR-p) and time integral values (SPR-i) [15].

#### 2.3.3. Lower Extremity Joint Angles

**Figure 1:**
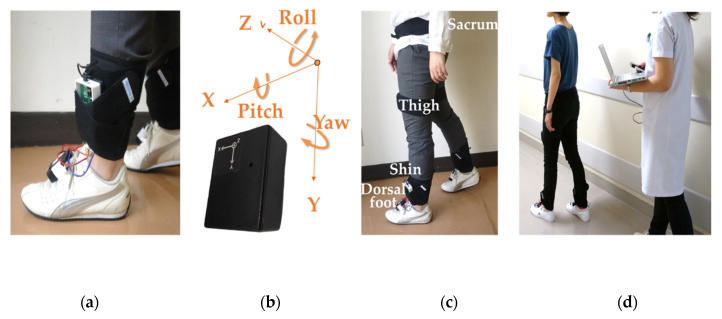
Attachment of sensors. (**a**) The Central Processing Unit (CPU) circuit board of the external force sensors was attached to the shin; (**b**) orientation of the motion sensors; (**c**) motion sensors were attached to the sacrum, thigh, shin, and dorsal foot; (**d**) the personal computer and CPU circuit board were connected wirelessly.

Lower extremity joint angles were measured using wireless motion sensors (LP-WSD1101-0A, Logical Product Corporation, Fukuoka, Japan). The motion sensors were attached to the sacrum, center of the thighs, center of the shins, and dorsal portion of the feet (Figure 1) and they were positioned at the *x*-axis + direction to the right and the *y*-axis + direction to the bottom. The sensor had a rectangular parallelepiped shape and was used in a different conventional way from the normal axis due to attaching problem. The *y*-axis indicated the vertical plane. The *y*-axis + direction was set downward on a known horizontal plane, it was measured for a few seconds and the initial value was set based on the average value, preventing noise from accumulating at each measurement. This procedure was automatically conducted by the software provided from the device company. The output of each motion sensor comprised three-dimensional geomagnetic, acceleration, and angular velocity data at the points. The measurement range of the acceleration sensor at the sacrum was ±5 g, and the angular velocity sensor was ±300 degrees per second; the measurement range of the acceleration sensor at the other sensors was ±5 g, and the angular velocity sensor was ±1500 degrees per second. The data were obtained at a 12-bit resolution.

All data were recorded at 100 Hz. The performances of these sensors were sufficient to measure walking accurately.

The three-axis relative angles were calculated using an angle calculation application (Oisaka Electronic Equipment Ltd., Fukuyama, Japan): the hip joint angle was measured using the data from the sacral and thigh sensors; the knee joint angle was obtained using the data from the thigh and shin sensors; and the ankle joint angle was derived using the data from the shin and dorsal foot sensors. Each of the acceleration and angular velocity datum of the internal motion sensor was integrated, and the limb angles with respect to the absolute coordinates were calculated using the Kalman filter. The relative angles were determined from those values. The acceleration and angular velocity were filtered using a low pass of 20 Hz. We used the geomagnetic data for correction. The accuracy of this calculation application was ±2%. To calculate the angle during walking, the minimum value was subtracted from the maximum value of each axis of joint angle [20].

#### 2.3.4. Footwear Characteristics

The participants were asked to wear their usual shoes when they visited the Diabetic Foot Outpatient Clinic. It was assumed that those shoes were most often worn during exercise therapy. The fitting length variable was calculated by subtracting the foot length from the inside length of the shoe. The fitting width variable was measured by subtracting the foot width from the inside width of the shoe.

The heel height (outsole thickness of the heel minus outsole thickness of the forefoot) of the shoes was measured using a tape measure. The hardness of the heel portion of the insole was measured three times using a durometer (Neutone, Try-All, Chiba, Japan), and the median of three measurements was considered. The measured value of the durometer had no unit, 50 was calculated as 1.70 ± 0.4 N, and 75 was calculated as 2.33 ± 0.5 N.

#### 2.3.5. Patient Characteristics

Data regarding age, sex, and height were obtained from medical records or by interview. Hallux valgus, bunionette, claw toe, and hammer toe were foot deformities and were identified by visual inspection by a well-trained nurse. Patients were diagnosed with diabetic neuropathy according to the diagnostic criteria of the American Diabetes Association [27]. Dorsiflexion/plantar flexion of the ankle joint and inversion/eversion of the subtalar joint were also evaluated. The passive range of motion was measured in the supine position using a goniometer [28]. Characteristics were examined for each leg. In other words, the characteristics data of each subject was analyzed twice. An interview was conducted on exercise habits.

### 2.4. Sample Size Calculation

The sample size calculation was based on the difference between two independent groups in our previous study using the G*Power software, version 3.1.9.2 [29]. The PSS of the second MTH of the six callus feet (96.1 ± 25.4 kPa) was determined to be higher than that of the 18 non-callus feet (63.5 ± 23.3 kPa) (*p* = 0.024) [23]. The effect size of 1.28, a α-error probability of 0.05, and a power (1-β error probability) of 0.80 were considered for the sample size calculation. Thus, the sample size of one group was estimated to be 11 feet in each group. Considering dropouts, the sample size of one group was set to 13 feet. Recruitment was continued until all groups had 13 feet.

### 2.5. Data Processing and Statistical Analysis

The variables of the external forces and foot motions were obtained by averaging 15 steps after removing the initial three steps and the final three steps. Data were extracted for each stance phase. Data cleaning (remove noises) was performed before analysis. All the data processing was performed using MATLAB R2012a (The Math Works, Inc., Natick, MA, USA).

The relationship of the external forces associated with callus formation and lower extremity joint angles, footwear characteristics, and patient characteristics were examined using Pearson’s correlation coefficient analysis. These variables were used for stepwise multiple-regression analysis for abs (r) > 0.100. When multicollinearity among the independent variables was observed—abs (r) > 0.600—with Pearson’s correlation coefficient analysis, only one of the variables was used in the model. Statistical analyses were performed using SPSS Statistics, version 23.0 (IBM, Chicago, IL, USA). Statistical significance was set at *p* = 0.05.

## 3. Results

During the observation period, 64 patients with diabetes visited the Diabetic Foot Outpatient Clinic, and 59 patients with diabetic neuropathy were included in the survey. All calluses had recurred by the time of the subsequent follow-up (1 month later); no callus was observed in any of the non-callus regions at follow-up. The patient and footwear characteristics appear in Table 1. The data were not normally distributed, tested using an F-test; all data were transformed logarithmically. As other results, there was no relationship between exercise habits and callus formation.

For the first MTH, the relationship between SPR-i and lower extremity joint angles, footwear characteristics, and patient characteristics was examined. Variables with a correlation coefficient of >0.1 included ankle abduction–adduction and flexion–extension angles, knee abduction–adduction, and flexion–extension angles, hip flexion–extension angle, 15-m walking time, sex, weight, body mass index (BMI), hemoglobin A1c (HbA1c), monofilament test (abnormal), angiopathy, toe-gap force, passive range of motion (dorsiflexion, plantar flexion, inversion, and eversion), fitting length and width, and hardness of insole. Data on hip flexion–extension angle and weight were excluded because of multicollinearity. The reason for excluding the hip flexion–extension angle was that the r value was smaller than the knee flexion–extension angle. Factors increasing SPR-i of the first MTH included small flexion–extension knee angle, large abduction–adduction ankle angle, soft insole, normality of monofilament test, and short 15-m walking time (Table 2). The adjusted R2 was determined to be 0.421.

For the second MTH, the relationship between SPR-i (which was associated with callus formation) and the lower extremity joint angles, footwear characteristics, and patient characteristics was similarly investigated. Variables with a correlation coefficient of >0.100 were as follows: ankle external rotation–internal rotation angle, 15-m walking time, sex, BMI, HbA1c, duration of diabetes, monofilament test (abnormal), toe-gap force, passive range of motion (dorsiflexion, eversion), fitting length, and hardness of insole. Data on weight were excluded because of multicollinearity. Factors increasing SPR-i of the second MTH included low HbA1c and high toe-gap force. The adjusted R2 was 0.227.

To determine the risk factors for callus formation in the second MTH, we conducted a further analysis among female patients as they constituted 88% of the callus formation group. The lower extremity joint angles and patient characteristics were compared between the female patients in the callus formation and non-callus formation groups (Table 3). Those in the callus formation group were found to have large ankle external rotation–internal rotation motion, large fitting length, low HbA1c, and high toe-gap force.

For the fifth MTH, the relationship between PSS (which was associated with callus formation) and the lower extremity joint angles, footwear characteristics, and patient characteristics was investigated. Variables with a correlation coefficient of >0.100 were identified as follows: ankle flexion–extension and external rotation–internal rotation angles, knee abduction–adduction angle, age, sex, weight, BMI, monofilament test (abnormal), history of diabetic foot ulcer, deformity, fitting length and width, and heel height. Data on weight were excluded because of multicollinearity. Factors increasing PSS of the fifth MTH included high BMI and tight fitting width (Table 4). The adjusted R2 was 0.146.

## 4. Discussion

This is the first study to identify the different risk factors of callus formation in the first, second, and fifth MTHs. If these relationships are examined, it may be possible to consider new interventions to lessen and prevent the pressure and shear stress associated with callus formation. The risk factors of high SPR-i in the first MTH included small flexion of the knee joint in terminal stance. Patients with such risk factors also had large pronation in foot motion during the push-off phase to obtain propulsion. Therefore, these motions might be improved to assist the push-off motion. SPR-i increased because the large abduction–adduction ankle angle was related to the small pressure [30]. In addition, regarding large abduction–adduction ankle motion, the ankle pronation motion during the push-off phase to obtain propulsion was considered. The ankle pronation motion was found to be compensated by the small flexion–extension knee angle. In our previous study, we found that subjects with callus (without distinguishing between callus regions) had small external rotation–internal rotation motion of body gravity [31]. This result may also indicate small propulsion. SPR-i increased since the soft insole off-loaded the pressure.

The normality of the monofilament test may indicate that complications of diabetes had not progressed, and the subjects’ usual daily activities were high because of normal perception. With high-level daily activities, external forces are applied on many occasions, and callus easily develops [32]. When the 15-m walking time was short (fast gait velocity), the external force increased by a stronger impact of the heel strike and push-off. The relationship between a short 15-m walking time and high pressure was recently clarified [33]. That study also considered increased abduction–adduction ankle motion for obtaining propulsion to maintain the short walking time.

In this study, no relationship was observed between SPR-i and lower extremity joint angles and footwear characteristics. As with the normal monofilament test result, low HbA1c and high toe-gap force may indicate that the complication of diabetes had not progressed, and the patients maintained high-level daily activities. Furthermore, only female patients were analyzed, because almost all calluses in the second MTH were formed in females. They had significantly large fitting length, large external rotation–internal rotation foot motion, low HbA1c, and high toe-gap force. It is conceivable that ankle external rotation–internal rotation angle increased to displace the plantar inside the footwear for long size footwear relative to the foot. In the current study, there was only one male subject, who had severe foot deformity, which may have resulted in outliers. Therefore, it is more likely that a patient who does not have severe foot deformity have the same tendency as the result of this time, rather than the result was obtained because it is a woman.

Factors increasing PSS of the fifth MTH included tight fitting width and high BMI. PSS and fitting width were found to have a negative correlation. It was considered that the higher shear stress of the fifth MTH was likely to occur if footwear was too tight. Shear stress could easily occur by fatty foot owing to high BMI.

From our results related to the first MTH, it is evident that interventions such as changing footwear and gait training should be considered. Regarding footwear, an intervention by assisting the push-off motion with rocker-sole footwear could be effective. A mild rocker-sole may be applicable for this purpose because it reduces the effort of walking, assists gait with increased propulsion, and provides metatarsal pressure relief [34]. Rocker-sole footwear may specifically improve push-off motion to prevent callus formation because such footwear slightly increases the motion of the lower limbs [35]. With respect to gait training, medical staff could suggest increasing the knee joint angle, thereby reducing pronation of the ankle joints during the push-off phase. Further research on these interventions is needed. Recent literature presents systems used for long acquisitions of gait measurement [36,37,38]. Using these systems, in addition to being able to measure a more natural gait, it will be possible to obtain a well gait by giving feedback when patients walk with appropriate joint angle.

For callus prevention of the second MTH, avoiding footwear that is too long may lead to small ankle external rotation–internal rotation angle, low SPR-i, and non-callus formation. Further study is required for interventions using appropriate size footwear.

For callus prevention of the fifth MTH, avoiding excessively tight footwear may lead to lower PSS. Further study is required regarding interventions with appropriate width footwear.

This study had two limitations. First, the patients in our survey were limited to those with mild neuropathy and mild foot deformity. Therefore, the results of this study may not apply to patients with progressive neuropathy, severe foot deformity, or amputation history. In future work, we would like to reveal the gait characteristics based on the severity of the foot condition.

Second, although the measurements were conducted after practice walking, walking in this research measured in a short distance. Thus, joint movements were generally smaller than in actual walking, and external forces may be smaller.

## 5. Conclusions

Callus has been identified as a non-ulcerative pathology in patients with diabetic neuropathy, and it can lead to severe diabetic foot ulcer. Thus, it is necessary to prevent callus recurrence.

In this study, we found that the risk factors of high SPR-i of the first MTH included small flexion of the knee joint in terminal stance. Patients with such risk factors also had large ankle pronation motion during the push-off phase to obtain propulsion. Thus, it is necessary to assist push-off so that propulsion can be obtained without using large ankle pronation motion. For the second MTH, patients with a risk of high SPR-i and callus formation were found to wear excessively long size footwear. For the fifth MTH, PSS was related to footwear with tight width. PSS was associated with callus formation in a previous study.

We conclude that for the first MTH, an effective intervention would involve assisting push-off foot motion to reduce SPR-i using rocker-sole footwear or gait training. For the second MTH, we suggest that avoiding overly long size footwear could prevent callus formation. For the fifth MTH, avoiding excessively tight footwear could prevent callus formation by lowering PSS.

In the future, it will be necessary to confirm the effect of preventing callus formation using appropriate footwear and gait training. Regarding footwear, an intervention by assisting the push-off motion with rocker-sole footwear could be effective.

## Figures and Tables

**Table 1:** Patient and footwear characteristics.

	1st Metatarsal Head			2nd Metatarsal Head			5th Metatarsal Head		
	Non-Callus Formation Group	Callus Formation Group	*p* Value	Non-Callus Formation Group	Callus Formation Group	*p* Value	Non-Callus Formation Group	Callus Formation Group	*p* Value
Number of feet	42 (76.4)	13 (23.6)		56 (77.8)	16 (22.2)		58 (73.4)	21 (26.6)	
Patient characteristics									
Age (years)	66.0 ± 11.2	63.7 ± 8.6	0.450 ^(1)^	66.7 ± 10.8	65.1 ± 7.8	0.517 ^(1)^	69.3 ± 10.2	67.0 ± 13.4	0.471 ^(1)^
Sex			0.053 ^(2)^			0.004 ^(2),^*			0.599 ^(2)^
Male	20 (47.6)	2 (15.4)		30 (53.6)	2 (12.5)		38 (65.5)	12 (57.1)	
Female	22 (52.4)	11 (84.6)		26 (46.4)	14 (87.5)		20 (34.5)	9 (42.9)	
Height (m)	1.61 ± 0.1	1.56 ± 0.1	0.133 ^(1)^	1.61 ± 0.09	1.57 ± 0.13	0.289 ^(1)^	1.62 ± 0.09	1.64 ± 0.13	0.504 ^(1)^
Weight (kg)	66.8 ± 17.5	66.9 ± 23.9	0.999 ^(1)^	68.0 ± 17.7	58.3 ± 9.3	0.005 ^(1),^*	63.1 ± 14.6	65.1 ± 14.8	0.595 ^(1)^
Body Mass Index	25.5 ± 5.4	26.8 ± 6.2	0.495 ^(1)^	25.9 ± 5.2	23.7 ± 3.9	0.068 ^(1)^	24.0 ± 4.4	24.2 ± 4.0	0.856 ^(1)^
Hemoglobin A1c (%)	6.9 ± 1.3	6.7 ± 0.7	0.134 ^(1)^	7.1 ± 1.0	6.3 ± 0.5	<0.001 ^(1),^*	6.7 ± 0.8	6.9 ± 1.2	0.507 ^(1)^
Diabetes duration (years)	15.6 ± 10.3	13.5 ± 7.5	0.683 ^(1)^	17.9 ± 9.9	13.4 ± 10.7	0.143 ^(1)^	15.7 ± 11.3	15.8 ± 7.1	0.986 ^(1)^
Monofilament test (abnormal)	5 (11.9)	1 (7.7)	1.000 ^(2)^	4 (7.1)	0 (0.0)	1.000 ^(2)^	5 (8.6)	4 (19.0)	0.236 ^(2)^
History of diabetic foot ulcer	2 (4.8)	0 (0.0)	1.000 ^(2)^	0 (0.0)	0 (0.0)		2 (3.4)	0 (0.0)	1.000 ^(2)^
Deformity	9 (21.4)	10 (76.9)	<0.001 ^(2),^*	7 (12.5)	7 (43.8)	0.010 ^(2),^*	8 (13.8)	5 (23.8)	0.314 ^(2)^
Angiopathy	2 (4.8)	1 (7.7)	0.562 ^(2)^	2 (3.6)	1 (6.3)	0.535 ^(2)^	4 (6.9)	1 (4.8)	1.000 ^(2)^
Toe-gap force (kgf)	1.8 ± 0.9	2.0 ± 0.8	0.561 ^(1)^	2.0 ± 0.7	2.3 ± 0.8	0.123 ^(1)^	2.1 ± 0.8	2.1 ± 0.7	0.839 ^(1)^
Dorsiflexion of ankle (°)	12 ± 8	12 ± 7	0.997 ^(1)^	11 ± 7	17 ± 9	0.029 ^(1),^*	12 ± 7	9 ± 10	0.142 ^(1)^
Plantar flexion of ankle (°)	42 ± 11	43 ± 8	0.868 ^(1)^	43 ± 7	49 ± 7	0.002 ^(1),^*	41 ± 6	43 ± 8	0.195 ^(1)^
Inversion of ankle (°)	43 ± 8	40 ± 10	0.366 ^(1)^	41 ± 9	44 ± 8	0.199 ^(1)^	41 ± 8	41 ± 10	0.863 ^(1)^
Eversion of ankle (°)	14 ± 7	15 ± 8	0.614 ^(1)^	15 ± 6	16 ± 6	0.434 ^(1)^	13 ± 5	13 ± 5	0.793 ^(1)^
Footwear characteristics									
Fitting length (cm)	0.9 ± 0.5	0.9 ± 0.5	0.925 ^(1)^	1.1 ± 0.9	1.0 ± 0.6	0.820 ^(1)^	1.2 ± 0.9	0.9 ± 0.8	0.312 ^(1)^
Fitting width (cm)	−0.4 ± 0.5	−0.3 ± 0.4	0.796 ^(1)^	−0.1 ± 0.5	−0.4 ± 0.5	0.043 ^(1),^*	−0.2 ± 0.5	−0.3 ± 0.4	0.367 ^(1)^
Heel height (cm)	1.7 ± 0.8	1.8 ± 0.7	0.539 ^(1)^	1.9 ± 0.9	2.3 ± 1.5	0.344 ^(1)^	1.7 ± 0.9	1.5 ± 0.5	0.323 ^(1)^
Hardness of insole	65 ± 9	65 ± 9	0.871 ^(1)^	65 ± 9	63 ± 10	0.665 ^(1)^	64 ± 10	62 ± 8	0.359 ^(1)^

Mean ± SD, *n* (%) * *p* < 0.05. ^(1)^ *t* test; ^(2)^ Fisher’s exact test.

**Table 2:** Relationship between the SPR-i (Shear stress Pressure Ratio of time integral value), lower extremity joint angles, and characteristics in the first metatarsal head.

	β (95% CI)	*p* Value	VIF
Knee flexion–extension angle (°)	−0.287 (−0.154, −0.018)	0.015	1.108
Hardness of insole	−0.494 (−0.152, −0.052)	<0.001	1.258
Monofilament test (abnormal)	−0.321 (−0.342, −0.048)	0.010	1.250
Ankle abduction–adduction angle (°)	0.239 (0.004, 0.095)	0.034	1.029
15-m walking time (sec)	−0.228 (−0.179, −0.002)	0.046	1.072

*n* = 55, Multiple regression analysis (stepwise selection). Input variables: ankle abduction–adduction and flexion–extension angle; knee abduction–adduction and flexion–extension angle; hip flexion–extension angle; 15-m walking time; sex; body mass index; hemoglobin A1c; monofilament test (abnormal); angiopathy; toe-gap force; passive range of motion (dorsiflexion, plantar flexion, inversion, and eversion); fitting length and width; and hardness of insole. Adjusted *R*^2^ = 0.421.

**Table 3:** Comparison of all data for the second metatarsal head in female patients.

	Non-Callus Formation Group	Callus Formation Group	*p* Value
Number of feet	26	14	
Lower extremity joint angles			
Ankle abduction–adduction angle (°)	49.6 ± 22.8	50.2 ± 20.8	0.937 ^(1)^
Ankle flexion–extension angle (°)	59.9 ± 20.9	67.6 ± 17.6	0.230 ^(1)^
Ankle external rotation–internal rotation angle (°)	36.3 ± 10.8	48.1 ± 19.2	0.049 ^(1),^*
Knee abduction–adduction angle (°)	26.7 ± 8.7	27.0 ± 11.9	0.934 ^(1)^
Knee flexion–extension angle (°)	30.3 ± 6.4	27.0 ± 8.3	0.208 ^(1)^
Knee external rotation–internal rotation angle (°)	30.6 ± 11.9	33.3 ± 13.3	0.527 ^(1)^
Hip abduction–adduction angle (°)	36.9 ± 14.7	44.5 ± 15.2	0.136 ^(1)^
Hip flexion–extension angle (°)	18.2 ± 4.0	20.1 ± 5.5	0.278 ^(1)^
Hip external rotation–internal rotation angle (°)	28.9 ± 16.3	32.2 ± 14.1	0.504 ^(1)^
15-m walking time (sec)	13.9 ± 1.7	11.8 ± 1.7	0.001 ^(1)^
Patient characteristics			
Age (years)	66.6 ± 11.6	65.2 ± 8.3	0.662 ^(1)^
Height (m)	1.54 ± 0.07	1.53 ± 0.04	0.515 ^(1)^
Weight (kg)	62.7 ± 16.9	56.6 ± 8.8	0.143 ^(1)^
Body Mass Index	26.2 ± 5.7	24.3 ± 3.8	0.210 ^(1)^
Hemoglobin A1c (%)	7.7 ± 1.0	6.4 ± 0.4	<0.001 ^(1),^*
Diabetes duration (years)	20.2 ± 7.8	15.0 ± 10.4	0.119 ^(1)^
Monofilament test (abnormal)	2 (7.7)	0 (0.0)	0.533 ^(2)^
History of diabetic foot ulcer	0 (0.0)	0 (0.0)	
Deformity	5 (19.2)	7 (50.0)	0.071 ^(2)^
Angiopathy	0 (0.0)	1 (7.1)	0.350 ^(2)^
Toe-gap force (kgf)	1.7 ± 0.6	2.3 ± 0.8	0.041 ^(1),^*
Dorsiflexion of ankle (deg)	12 ± 7	15 ± 7	0.316 ^(1)^
Plantar flexion of ankle (deg)	47 ± 8	48 ± 6	0.449 ^(1)^
Inversion of ankle (deg)	45 ± 8	45 ± 8	0.891 ^(1)^
Eversion of ankle (deg)	16 ± 7	16 ± 7	0.980 ^(1)^
Footwear characteristics			
Fitting length (cm)	0.5 ± 0.6	0.9 ± 0.6	0.026 ^(1),^*
Fitting width (cm)	−0.2 ± 0.5	−0.5 ± 0.5	0.103 ^(1)^
Heel height (cm)	2.3 ± 1.0	2.4 ± 1.5	0.855 ^(1)^
Hardness of insole	66 ± 8	65 ± 10	0.717 ^(1)^

Mean ± SD, *n* (%) * *p* < 0.05. ^(1)^ *t* test; ^(2)^ Fisher’s exact test.

**Table 4:** Relationship between PSS (peak shear stress) and, lower extremity joint angles and characteristics in the fifth metatarsal head.

	β (95% CI)	*p* Value	VIF
Fitting width (cm)	−0.305 (−2.021, −0.381)	0.005	1.000
Body Mass Index	0.274 (0.099, 0.725)	0.011	1.000

*n* = 79, Multiple regression analysis (stepwise selection). Input variables: ankle flexion–extension and external rotation–internal rotation angle; knee abduction–adduction angle; age; sex; weight; Body Mass Index; monofilament test (abnormal); history of diabetic foot ulcer; deformity; fitting length and width; and heel height. Adjusted *R*^2^ = 0.146. VIF: variance inflation factor.
